# Comparison of numerical and standard sarnat grading using the NICHD and SIBEN methods

**DOI:** 10.1038/s41372-021-01180-w

**Published:** 2021-08-14

**Authors:** Brian H. Walsh, Chelsea Munster, Hoda El-Shibiny, Edward Yang, Terrie E. Inder, Mohamed El-Dib

**Affiliations:** 1 Department of Neonatology, Cork University Maternity Hospital, Cork, Ireland; 2 Department of Pediatric Newborn Medicine, Brigham and Women’s Hospital, Boston, USA; 3 Department of Radiology, Boston Children’s Hospital, Boston, USA

**Keywords:** Paediatric neurological disorders, Paediatrics, Physical examination

## Abstract

**Objective:**

The NICHD and SIBEN assessments are adapted from the Sarnat grade, and used to determine severity of neonatal encephalopathy (NE). We compare NICHD and SIBEN methods, and their ability to define a minimum threshold associated with significant cerebral injury.

**Study design:**

Between 2016 and 2019, 145 infants with NE (77-mild; 65-moderate; 3-severe) were included. NICHD and SIBEN grade and numerical scores were assigned. Kappa scores described agreement between methods, and ROC curves their ability to predict MR injury.

**Results:**

Good agreement existed between grading systems (*K* = 0.86). SIBEN defined more infants as moderate, and less as mild, than NICHD (*p* < 0.001). Both numerical scores were superior to standard grades in predicting MR injury.

**Conclusion:**

Despite good agreement between methods, SIBEN defines more infants as moderate NE. Both numerical scores were superior to standard grade, and comparable to each other, in defining a minimum threshold for cerebral injury. Further assessment contrasting their predictive ability for long-term outcome is required.

## Introduction

The Sarnat grade, and subsequent modifications of it, are used to define the severity of Neonatal Encephalopathy (NE), and are predictive of cerebral injury as well as long-term outcome [1, 2]. However to be used appropriately a grade of encephalopathy cannot be assigned for several days using these scoring systems, long after the window for treatment with therapeutic hypothermia (TH) has elapsed. In recognition of this, early grading systems derived from the Sarnat grade have been developed, to allow timely assessment of NE and determination of TH eligibility. However, recent studies have shown that infants with milder encephalopathy may still demonstrate an increased risk for cerebral injury challenging clinicians to review the systematic neurological assessment used to evaluate these infants [3–5]. The goal for clinicians should now be to determine how best to identify the minimum threshold at which injury occurs.

The NICHD system is commonly used in North America to define severity of encephalopathy into one of three grades, mild, moderate or severe [6, 7]. However severity of encephalopathy is not as simple as these grades. In the recent PRIME study, Chalak et al. applied a numerical value (0–3) to each of the six domain of the NICHD system in their cohort of infants with mild NE [7, 8]. Using this method they demonstrated that there is a range of severity within each grade of encephalopathy, and that the distinction between grades is often vague, with overlapping values when a numerical score is applied. For example infants with mild NE could score from 1 to 10, while moderate NE ranged from 6 to 14, and severe NE from 9 to 18. Perhaps unsurprisingly infants with mild NE that had higher NICHD scores (≥5) were those at risk of neuro-developmental impairment.

The SIBEN system has recently been described as an alternate method of early assessment [7, 8]. The SIBEN exam assesses the same domains as the NICHD system, defining the severity of encephalopathy as mild, moderate or severe. However unlike the NICHD system, SIBEN does not weight either seizures, or the sub-domains of the primitive reflexes and autonomic system. Each of these findings is counted as an equal domain. Therefore although the same exam is performed, SIBEN requires findings in ≥3 out of ten domains, while NICHD requires findings in ≥3 out of six domains to categortize an infant as moderate or severe NE. It is likely that this would result in some infants being defined as moderate NE by SIBEN, but mild by NICHD, although the two methods have not been previously compared to confirm this.

In this manuscript our aim was to assess the variation of encephalopathy severity described by numerical scoring systems applied to both the NICHD and SIBEN grading systems, and to determine if either system (using both grades and numerical scores) was superior at detecting a minimum threshold associated with significant cerebral perturbation or injury, defined by the development of electrographic seizures, abnormal electroencephalography (EEG) background at 24 h, or significant MRI injury.

## Methodology

This is a retrospective analysis of infants with NE who underwent TH between January 2016 and May 2019 in the Brigham and Women’s Hospital, a large tertiary level NICU. The need to initiate TH was determined by the clinician, and based upon an adaptation of standard criteria which we have previously described [5, 9]. Specifically these criteria state and infant must be (1) >34 weeks gestation, and (2) have a risk for perinatal asphyxia defined as one of the following; sentinel event prior to delivery (e.g. uterine rupture); or prolonged resuscitation at birth (on-going intermittent positive pressure ventilation or intubated at 10 min after birth); or 10 min Apgar score ≤5; or pH ≤7.1 from the cord gas or postnatal gas within the first 1 h of life; or base deficit ≥10 mEq/L from the cord gas or postnatal gas within the first 1 h of life; and (3) evidence of seizures or clinical encephalopathy. In assessing pH and base deficit, while the umbilical arterial values are most reflective of the fetal state [10], the worst value from any cord or postnatal sample in the first hour of life was used to determine TH eligibility in accordance with the methodology of the TH RCTs and international recommendations [11–16]. Available samples included umbilical arterial samples in 120 cases, umbilical venous in 124 cases (paired umbilical arterial and venous in 117), and postnatal blood gas samples in 140. Of the worst samples reported—116 were umbilical arterial (4 arterial samples were excluded as the results appeared erroneous when compared to the cord venous and or postnatal samples), 6 were umbilical venous (cases with no umbilical arterial sample present), and 23 were postnatal samples. All infants had a neurological exam performed by both an attending neonatologist and a pediatric neurologist, within the first 6 h of life prior to initiation of TH. For inclusion in this analysis, in addition to assigning an overall grade of NE, all components and sub-components of the NICHD exam had to be documented in-full within the medical records. Institutional Review Board approval was obtained to conduct this analysis from the Partners Human Research Committee.

Demographic, clinical and laboratory data, including maternal prenatal history, delivery history, and postnatal history until discharge, were collected from the medical records. All infants included in this cohort underwent TH, had multichannel EEG during cooling and had an MRI scan following re-warming. The continuous EEGs were placed as soon as possible after the decision to initiate TH, and were maintained through-out the 72 h of cooling and re-warming. For this analysis the clinical neurophysiologist report from the first 24 h of age were used to define the EEG grade of encephalopathy. The presence of electrographic seizures at any point during the EEG monitoring was recorded.

### NICHD grade and score

The NICHD grade assesses six neurological domains; level of consciousness, spontaneous activity, tone, posture, primitive reflexes (two sub-domains assessed independently, with the worst score providing the global grade for primitive reflexes- suck, and Moro reflex), and autonomic activity (three sub-domains assessed independently, with the worst score providing the global grade for autonomic activity, pupillary reaction, heart rate and respirations) (Supplementary Table 1). Each of the six domains is defined as normal, mild, moderate or severe. A global grade of NE is then determined; an infant is scored as mild if they have at least one domain consistent with mild, but do not meet criteria for moderate or severe NE; an infant is scored as moderate if they have three or more domains consistent with moderate or severe NE, but more domains are moderate than severe; and they are scored severe if they have three or more domains consistent with moderate or severe NE, but more domains are severe than moderate. Seizures are not included in the six domains, however the presence of seizures and findings of NE automatically define the grade of NE as at least moderate.

The NICHD score described by Chalak et al. assigned a numerical score from 0 to 3 (consistent with normal to severe findings respectively) to each of the six domains. The score across all domains was then added and a global numerical score provided [7]. Seizures were not included in this system.

### SIBEN grade and score

The SIBEN grade assesses the same exam components as the NICHD system; level of consciousness, spontaneous activity, tone, posture, suck reflex, Moro reflex, pupillary reaction, heart rate and respirations (Supplementary Table 1) [6]. However it does not weight the sub-domains of the autonomic system or primitive reflexes, and each is treated as an equal independent domain. Additionally seizures are counted within the score, as an independent domain. Of note seizures do not automatically assign a global grade of moderate NE. Therefore there are ten domains in the SIBEN system, each of which is defined as normal, mild, moderate or severe. A global grade of NE is then determined; an infant is scored as mild if they have at least three domains consistent with mild, but do not meet criteria for moderate or severe NE; an infant is scored as moderate if they have three or more domains consistent with moderate or severe NE, but more domains are moderate than severe; and they are scored severe if they have three or more domains consistent with moderate or severe NE, but more domains are severe than moderate. Of note, seizures were included in defining grade if they occurred in the first 6 h of life. If they occurred at a later point, the grade was not revised due to the development of seizures.

A numerical score has not previously been published for the SIBEN system. We have therefore proceeded in a similar fashion to that described by Chalak et al. assigning a numerical score from 0 to 3 (consistent with normal to severe findings respectively) to each of the ten domains. The score across all domains was then added and a global numerical score provided.

### Magnetic resonance imaging

All infants had at least one cranial MRI performed within the first week of life. The clinical teams caring for the infant determined the timing of these MRI studies and whether a second MRI was required. Only the initial MRI scans are reported here due to the variability in a second scan being performed. All scans were performed on a 3-T Siemens scanner (Siemens, Erlangen, Germany). The standard clinical imaging protocol included T1, T2, and diffusion weighted imaging. The images for this study were analyzed independently by a pediatric neuroradiologist, and neonatologist (EY, TI), who were blinded to the clinical grades of encephalopathy. The presence and type of any MRI abnormalities were detailed. Analysis of the pattern and severity of brain injury was classified according to the grading system developed by Barkovich et al., which has been extensively validated in NE using both conventional and diffusion weighted sequences [17]. A score of ≥2 in the deep nuclear gray matter, or a score of ≥3 in a watershed pattern, was considered consistent with moderate-severe MRI injury.

### Statistical analyses

Statistical analysis was performed using PASW statistics 18.0. Nonparametric data were reported as median values with inter-quartile range (IQR) or range as specified in the text, and comparisons performed using the Mann–Whitney *U* test or Kruskal–Wallis *H* Test, as appropriate. The Chi Squared test was used when comparing proportions. Agreement between grades was described using Kappa values.

Logistic regression analysis was performed to assess the strength of association between the individual methods of assessing NE severity (covariate) and the development of moderate-severe MR cerebral injury (dependent variable). Additionally receiver operator curves were generated to determine their ability to predict moderate-severe MR cerebral injury. The sensitivity and specificity were determined from the ROC curves. We wished to determine the benefits of these methods to be used as a screening tool to detect the minimum threshold for significant injury. Therefore the cut-off value described was that associated with the optimum sensitivity. Statistical significance was taken as *p* < 0.05, and a Bonferroni correction was applied for post-hoc sub-group analysis.

## Results

During the study period there were 170 infants with NE who received TH in our center. Twenty five infants were excluded as they had incomplete documentation of the NE assessment sub-domains (Of those exclude 11 were documented as having mild NE, 9 moderate NE, 2 with severe NE, and 2 did not have any grade of NE assigned). This left a study population of 145 infants. Demographic details are provided in Table 1.

**Table 1:** Clinical and demographic details for cohort.

NICHD grade	Mild NE (*n* = 77)	Moderate NE (*n* = 65)	Severe NE (*n* = 3)
Gestational age	39.14 (1.58)	38.83 (1.77)	40.7 (1.12)
Birth weight	3232 (535)	3085 (532)	3656 (390)
Sex (female)	34 (44%)	25 (38.5%)	1 (33%)
Method of delivery			
SVD	23 (30%)	23 (35%)	
Instrumental	10 (14%)	10 (15%)	2 (66%)
Em-LSCS	39 (51%)	31 (48%)	1 (33%)
El-LSCS	4 (5%)	1 (2%)	
pH^a^	7.02 (6.80–7.35)	7.04 (6.80–7.35)	6.94 (6.80–7.23)
Base deficit^a^	12.9 (10.6–14.9)	13 (10.2–13.4)	17.6 (16.4–18.8)
Apgar 5 min	7 (2–9)	6 (0–8)	0 (0–2)
Apgar 10 min	8 (5–10)	7 (0–10)	2 (1–3)
Invasive ventilation	13 (17%)	23 (35%)	3 (100%)
Electrical seizure	7 (9%)	9 (14%)	3 (100%)
Seizure 1st 6 h	0 (0%)	1 (2%)	0 (0%)
EEG background			
Normal	14 (18%)	13 (20%)	0
Mild	52 (68%)	31 (48%)	0
Moderate	7 (9%)	10 (15%)	0
Severe	0	1 (2%)	3 (100)
Length of stay	7 (4–42)	8 (2–35)	18 (9–38)

^a^Lowest value from either an umbilical cord blood sample or any postnatal blood sample <1 h after birth. (Sample used—umbilical cord artery *n* = 116, umbilical vein *n* = 6, and postnatal sample *n* = 23).

### NICHD grade and numerical score

**Fig. 1:**
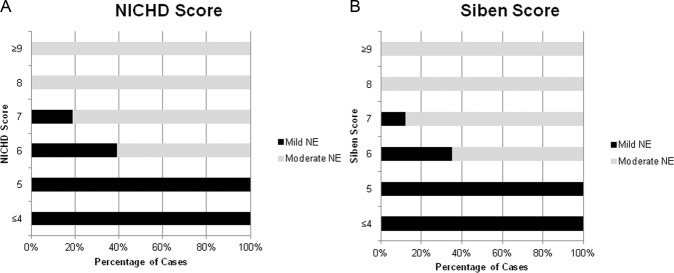
Association between Grade of Encephalopathy and Numerical Score. **A** Frequency of NICHD Grade associated with specific NICHD Numerical Score. **B** Frequency of SIBEN Grade associated with specific SIBEN Numerical Score.

Of the 145 infants, based upon the NICHD criteria, 77 infants had mild NE, 65 had moderate, and 3 had severe NE. There was a significant difference in the median NICHD Numerical score between those with mild or moderate NE in our cohort, 5 (range 1–7) vs. 8 (range 6-13) vs. 15 (range 15–18), *p* < 0.001, respectively. Figure 1 demonstrates that there is a transition in scores between mild and moderate NE in the 6–10 range as expected. As can be seen in our data, although possible for a mild infant to have a score of 9 or 10, no mild NE had a score above 7. Of our cohort, 16% [12] of mild NE, and 42% (27) of infants with moderate NE had a score within the overlapping range of 6–7.

### SIBEN grade and numerical score

Utilising the SIBEN system 68 had mild NE, 74 had moderate NE, and 3 had severe NE. Similar to the NICHD score, there was a significant difference in the median SIBEN scores between grades, with a median score of 4 (range 1–7) vs. 8 (range 6–17) vs. 19 (range 18–22), *p* < 0.001, respectively. Again there was overlap between mild and moderate NE grades, with 13% (*n* = 9) and 38% (*n* = 28), of the SIBEN mild and moderate NE infants, respectively, having a score from 6 to 7 (Fig. 1).

### Comparison of NICHD and SIBEN grade

The NICHD and SIBEN grading systems agreed in 92% of cases (134/145), with a Kappa value of 0.86 (*p* < 0.001). Despite this high rate of agreement, the differences between the assigned grades was statistically significant (*p* < 0.001), with the SIBEN system having fewer infants defined as mild NE, and more as moderate NE, compared to the NICHD system. Details on all infants for whom there was a discrepancy in the grades is provided in Table 2. As can be seen, the majority are infants with mild NE by the NICHD but moderate using SIBEN, with NICHD scores of <6. The only infant that was moderate per NICHD but mild per SIBEN, had hypertonia in addition to two other moderately abnormal domains. Hypertonia is not included on the SIBEN score, hence the reason for the discrepancy in grades.

**Table 2:** Details of infants with seizures, or discrepancy in grades between systems.

	NICHD grade	SIBEN grade	NICHD score	SIBEN score	EEG 24 h	EEG seizures >6 h age	Descriptive MRI findings	Barkovich BGT score	Barkovich WS score	Barkovich overall grade
1	Mild	Moderate	4	6	Normal	No	Normal	0	0	Normal
2	Mild	Moderate	4	6	Mild	No	Normal	0	0	Normal
3	Mild	Moderate	4	6	Mild	No	Cerebellar hemorrhage	0	0	Normal
4	Mild	Moderate	5	7	Mild	No	Normal	0	0	Normal
5	Mild	Moderate	5	7	Moderate	Yes	Bilateral parasagital injury to white mater and cortex (not involving peri-rolandic cortex)	0	4	Moderate-severe
6	Mild	Moderate	5	7	Normal	No	Sub-dural hematoma	0	0	Normal
7	Mild	Moderate	6	8	Mild	No	Normal	0	0	Normal
8	Mild	Moderate	6	8	Mild	No	Normal	0	0	Normal
9	Mild	Moderate	7	9	Mild	No	Normal	0	0	Normal
10	Mild	Moderate	7	9	Normal	No	Increased signal bilaterally in posterior watershed white mater, hippocampus, and cerebellar hemorrhage	0	2	Mild
11	Moderate^a^	Mild	7	5	Mild	No	Normal	0	0	Normal

^a^Infant had hypertonicity, plus 2 other domains with findings consistent with Moderate NE. Hypertonicity not part of SIBEN grade- therefore Moderate NE by NICHD (3 moderate domains), but Mild NE by SIBEN (2 moderate domains).

### Association with EEG

**Fig. 2:**
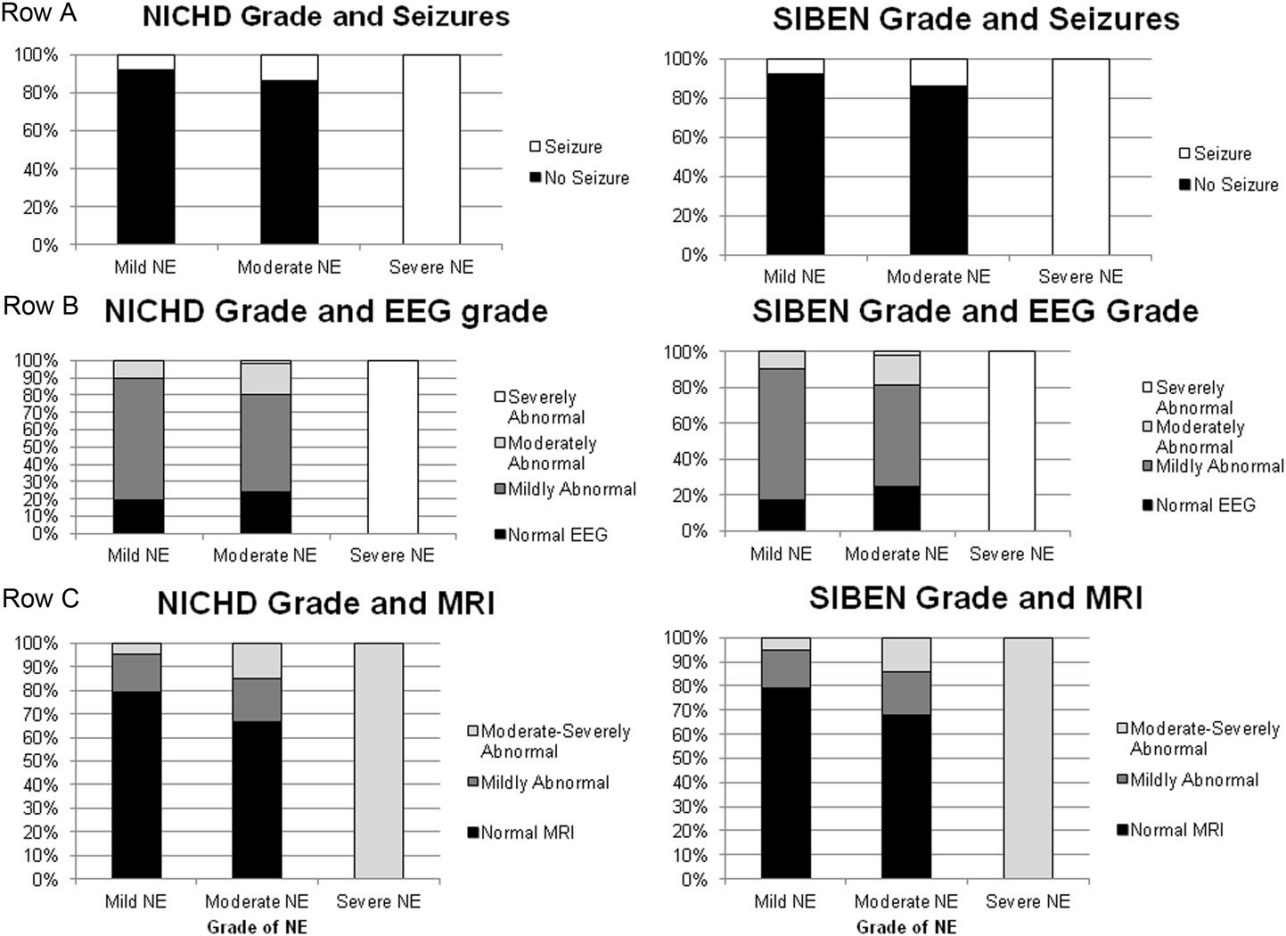
Association between NICHD and SIBEN grades and short-term outcomes. **A** A It demonstrates association of NICHD and SIBEN grade with presence of Seizures. **B** It demonstrates association of NICHD and SIBEN grade with multi-channel EEG grade. **C** It demonstrates association of NICHD and SIBEN grade with MRI cerebral injury.

By 24 h of age, the background EEG was normal in 27 infants, mildly encephalopathic in 83, moderately encephalopathic in 17 and severely encephalopathic in 4. EEG data was not available on 14 infants. Nineteen infants developed electrographic seizures. The median age of seizure onset was 13 h (IQR 7–17 h). The distribution of background EEG grade, and electrographic seizures for each of the grading systems is demonstrated in Fig. 2.

In comparing the systems, there was no difference in the background EEG grade for those classified as mild per NICHD vs. mild per SIBEN (*p* = 0.95), moderate per NICHD vs. moderate per SIBEN (*p* = 0.97) or severe per NICHD vs. severe per SIBEN (*p* = 1) NE. Seven infants that were graded as mild per NICHD versus 6 who were mild per SIBEN developed electrographic seizures (*p* = 0.59). Similarly there was no difference in frequency of electrographic seizures between those classified using the NICHD vs. SIBEN system as moderate (*p* = 0.99), or severe (*p* = 1) NE.

### Association of early neurological assessment with cerebral MRI

The median age at MRI scan was 4 (range 0–15) days. 104 (72%) MRIs had no findings consistent with ischemic injury on the Barkovich system, while 41 (28%) did demonstrate ischemic injury. Of these 41 with identified injury, 15 had injury within the Basal Ganglia and/or Thalamus, and 34 had injury in a Watershed distribution. Twenty four (17%) infants MRIs’ were consistent with mild injury, and 17 (12%) had moderate or severe cerebral injury on MRI. The distribution of cerebral MR injury stratified by each of the grading systems is demonstrated in Fig. 2. Although some variation between the grading systems, there was no significant difference in the frequency or severity of MR cerebral injury comparing those classified using the NICHD vs. SIBEN grade as mild (*p* = 0.97), moderate (*p* = 0.92) or severe (*p* = 1) NE.

Logistic regression was used to compare the strength of association between, while receiver operator curves were generated to assess predictive ability for, each of the methods of neurological assessment (NICHD grade, SIBEN grade, NICHD score, and SIBEN score) and a moderate-severely abnormal MRI (Table 3). As can be seen each method significantly predicted the development of a moderate-severely abnormal MRI. The NICHD and SIBEN numerical scoring systems had a greater association, and were superior at predicting a moderate-severely abnormal MRI than the categorical grades using either system. There was no difference in the strength of association between the NICHD and SIBEN numerical scores. For both of the scoring systems a cut-off of ≥4 was the optimum score for identifying those with a moderate-severe cerebral injury on MRI (Table 3).

**Table 3:** Ability of each grading and scoring system to predict Moderate-Severe Hypoxic Ischemic injury on MRI.

To predict a moderate-severe MRI								
	*r*^2^	*p*	AUC	*p*	CI	Cut-off	Sensitivity	Specificity
NICHD grade	0.221	<0.001	0.71	0.006	0.57–0.84	Moderate	76	57
SIBEN grade	0.227	<0.001	0.71	0.005	0.58–0.84	Moderate	78	49
NICHD score	0.267	<0.001	0.79	<0.001	0.67–0.90	≥4	100	9
SIBEN score	0.260	<0.001	0.78	<0.001	0.66–0.89	≥4	100	10

## Discussion

In this study we report that while there is good agreement between the NICHD and SIBEN systems, there are subtle but important differences. The disagreements between the two occur at the threshold between mild and moderate NE. This reflects the fact that within an individual grade of NE there is a range of severity. The use of numerical scoring systems highlights this issue, documenting the range within each grade, and the considerable overlap in the burden of neurological disturbance observed between grades. We report that both the NICHD and SIBEN grading systems detected those at increased risk of significant cerebral injury, however numerical scoring systems for either method were superior for detecting the minimum threshold of increased risk. The numerical scoring systems were comparable, and for both we found a cut-off of ≥4 provided the optimum sensitivity for detecting a significant cerebral insult.

There was over 90% agreement between the NICHD and SIBEN grades assigned. The differences in the grades are primarily due to the differing methods of synthesizing the same exam findings [6, 7]. Unlike the NICHD system, SIBEN does not weight the autonomic system (heart rate, respiration, pupils) and primitive reflexes (suck, Moro) by category, but counts each of the five sub-domains equally. Therefore for NICHD the worst finding from the constituent sub-domains are used to assign a single grade to both the autonomic system and primitive reflexes, but for SIBEN 5 equal grades are defined. While SIBEN’s approach is in keeping with the original Sarnat method, the NICHD’s approach would ensure a broader neurological disturbance is present when defining moderate or severe NE, which would reduce the numbers of cases. This reflects our findings, that the SIBEN system assigned several infants as moderate NE, that were classified as mild NE by the NICHD system.

In this manuscript, we contrast two numerical scoring systems. The NICHD score was recently described by Chalak et al. and the second numerical score based upon the SIBEN grade has not previously been published. Alternate numerical scores have been described for prognosis following NE, however unlike the NICHD and SIBEN methods, these scores were designed to predict long-term prognosis over several days, rather than to be used in the first hours of life to identify encephalopathy and determine treatment eligibility [18, 19]. Furthermore, several of the domains described in these scores differ between those of the NCIHD and SIBEN methods, and include assessments of fontanel, grasp, and feeding, which may not be practical within the first 6 h [18, 19]. Potentially due to such issues, they have been reported to poorly identify those eligible for TH [20]. In contrast we report that both numerical scoring systems were superior to the standard grade of encephalopathy for predicting which infants would develop significant cerebral perturbation and injury.

Numerical scoring systems have several potential advantages compared to standard grading systems. Chalak et al. use the NICHD score to demonstrate the range of severity that exists among those with mild NE [7]. Unlike the work of Chalak et al., our data includes infants with mild, moderate and severe NE. This has allowed us to demonstrate the range in severity, and overlap, that exists particularly among those with mild and moderate NE. Using, the NICHD score 16% of mild and 42% of moderate grades had overlapping scores in the 6–7 range (NICHD score mild 1–10, moderate 6–14). The overlap was slightly less using the SIBEN score, with 13% of mild and 38% of moderate cases having overlapping scores. Being able to assess the severity within an individual grade of NE is necessary to highlight those at greater risk outside of the classical TH eligibility, such as those with mild NE at greater risk of injury.

An additional benefit of numerical scoring systems is that they reduce the impact of several of the more subjective components of either the NICHD or SIBEN exam. For example a weak suck can be defined as a mild or moderately encephalopathic finding using either assessment method. There is similar overlap in findings between mild and moderate encephalopathy for assessment of tone, and spontaneous activity. These overlapping findings blur the distinction between grades, and allow some subjectivity in assessment. It must be recognized that this blurring of findings between grades is not necessarily inaccurate, and in fact likely reflects the true nature of this disease which is a continuum rather than distinct isolated grades [1]. However the subjectivity that they introduce can be problematic when so much emphasis is placed on the grade of NE for determining eligibility for TH. The use of a numerical scoring system rather than grade of NE to determine risk of injury would limit the impact of these more subjective components of the exam, as for example with the NICHD system a difference in 1 or 2 points when being assessed on a continuous scale from 0 to 18 would have less of an impact on the over-all synthesis.

The primary limitation of this study is that there is no long-term neuro-developmental outcome data available for this cohort. Although this is a limitation, cerebral injury on MRI is highly associated with 2 year neuro-developmental outcome [21], with the presence of moderate-severe injury being highly predictive of significant neuro-developmental impairment. Furthermore it must be recognized that the SIBEN grade itself has not yet been validated for neuro-developmental outcome. Given it’s similarities with the NICHD grade, and the similar results for predicting a significantly abnormal MRI that we report here, it is likely that it will have similar predictive abilities as the NICHD grade for long-term outcome, but this is yet to be validated. An additional limitation is that this was a secondary analysis. As such it is retrospective in nature, with the potential for unidentified confounders impacting the results. However the fact that this is a large cohort of infants with NE, including 142 infants with mild or moderate NE, somewhat reduces such risk. Lastly, this study was limited by the fact that our local guidelines include providing TH to those with milder grades of encephalopathy [5, 9]. As such all infants included in the current analysis underwent TH. It is unknown if TH reduces the burden of injury with mild NE, however there is evidence of improved MR spectroscopy in these infants [11, 22]. Twenty eight percent of infants in our study demonstrated cerebral injury on MRI. This is consistent with recent literature, which has demonstrated that the majority of those with moderate or severe NE that undergo TH have a normal MRI scan [23]. Rollins et al. recently reported that only 17% of those with moderate or severe NE have a significant MR injury following TH [24]. Therefore treatment with TH may have adjusted the optimum cut-offs for the numerical scores to identify injury. This will need to be studied further in infants that do not receive TH.

In conclusion we found a strong agreement between the SIBEN and NICHD systems. The SIBEN did define more infants as moderate NE than the NICHD system did. Numerical scores based upon both systems highlighted the range of neurological disturbance that exists within individual grades of NE. Likely for this reason the numerical scores were superior at identifying infants at increased risk of developing seizures, significant EEG abnormalities and MRI injury. We propose that screening for TH eligibility should be determined using numerical scores rather than grade of NE. This would aid in identifying those most at risk of injury, particularly for those at the threshold between grades who do not currently receive therapy. To adopt such an approach further work is required to validate the minimum threshold for injury among those who do not receive TH.

## Supplementary information

Supplemental Table 1
