# Supplementary material for: Comparison of numerical and standard sarnat grading using the NICHD and SIBEN methods
Source: J Perinatol. 2021 Aug 14;42(3):328–34. doi: 10.1038/s41372-021-01180-w (PMC8913366; doi:10.1038/s41372-021-01180-w)
Supplement: Supplementary file 1 — Supplemental Table 1 [file 41372_2021_1180_MOESM1_ESM.doc]

**Supplemental Table 1:** NICHD and SIBEN grading systems, with equivalent numerical scores presented

|  | **Mild NE** | **Moderate NE** | **Severe NE** |
| --- | --- | --- | --- |
|  |  |  |  |
| **Level of Consciousness** |  |  |  |
| NICHD | 1=Hyper-alert | 2=Lethargic | 3=Stupor/Coma |
| SIBEN | 1=Hyper-alert | 2=Lethargic | 3=Stupor/Coma |
| **Spontaneous Activity** |  |  |  |
| NICHD | 1=Normal/decreased | 2=Decreased | 3=Absent |
| SIBEN | 0=Normal | 2=Decreased | 3=Absent |
| **Muscle tone** |  |  |  |
| NICHD | 1=Normal/slight increase | 2a=Hypotonia  2b=Hypertonia | 3a=Flaccid  3b=Rigid |
| SIBEN | 0=Normal | 2=Hypotonia | 3=Flaccid |
| **Posture** |  |  |  |
| NICHD | 1=Mild distal flexion | 2=Distal flexion complete extension | 3=Decerebrate |
| SIBEN | 1=Mild distal flexion | 2=Marked distal flexion | 3=Decerebrate |
| **Primitive Reflexes** | | | |
| **Suck** |  |  |  |
| NICHD | 1=Weak or Incomplete | 2=Weak or Incomplete and/or bite | 3=Absent |
| SIBEN | 1=Weak | 2=Weak or absent | 3=Absent |
| **Moro** |  |  |  |
| NICHD | 1=Intact; low threshold | 2=Incomplete | 3=Absent |
| SIBEN | 1=Strong | 2=Weak | 3=Absent |
| **Autonomic Function** | | | |
| **Pupils** |  |  |  |
| NICHD | 1=Mydriasis | 2=Miosis | 3=Variable/non-reactive |
| SIBEN | 1=Mydriasis | 2=Miosis | 3=Diverted/non-reactive |
| **Heart Rate** |  |  |  |
| NICHD | 1=Tachycardia | 2=Bradycardia | 3=Variable |
| SIBEN | 1=Tachycardia | 2=Bradycardia | 3=Variable |
| **Respiration** |  |  |  |
| NICHD | 1=Hyperventilation | 2=Periodic Breathing | 3=Apnea or Needing Ventilation |
| SIBEN | 0=Normal | 2=Periodic Breathing | 3=Apnea |
| **Seizures** |  |  |  |
| NICHD* | N/A | N/A | N/A |
| SIBEN | 0=Absent | 2=Frequent | 3=Infrequent |

*Not part of criteria for grading but if present and mild NE criteria are defined as moderate NE
